# Pattern Recognition of the COVID-19 Pandemic in the United States: Implications for Disease Mitigation

**DOI:** 10.3390/ijerph18052493

**Published:** 2021-03-03

**Authors:** Jianyong Wu, Shuying Sha

**Affiliations:** 1 Data Explorer LLC, Chapel Hill, NC 27514, USA; 2 Gillings School of Global Public Health, University of North Carolina, Chapel Hill, NC 27599, USA; 3 School of Nursing, University of Louisville, Louisville, KY 40202, USA; s0sha002@louisville.edu

**Keywords:** coronavirus, machine learning, K-means clustering, seasonal-trend decomposition, spatial pattern

## Abstract

The novel coronavirus (COVID-19) pandemic presents a severe threat to human health worldwide. The United States (US) has the highest number of reported COVID-19 cases, and over 16 million people were infected up to the 12 December 2020. To better understand and mitigate the spread of the disease, it is necessary to recognize the pattern of the outbreak. In this study, we explored the patterns of COVID-19 cases in the US from 1 March to 12 December 2020. The county-level cases and rates of the disease were mapped using a geographic information system (GIS). The overall trend of the disease in the US, as well as in each of its 50 individual states, were analyzed by the seasonal-trend decomposition. The disease curve in each state was further examined using K-means clustering and principal component analysis (PCA). The results showed that three clusters were observed in the early phase (1 March–31 May). New York has a unique pattern of the disease curve and was assigned one cluster alone. Two clusters were observed in the middle phase (1 June–30 September). California, Texas and Florida were assigned in the same cluster, which has the pattern different from the remaining states. In the late phase (1 October–12 December), California has a unique pattern of the disease curve and was assigned a cluster alone. In the whole period, three clusters were observed. California, Texas and Florida still have similar patterns and were assigned in the same cluster. The trend analysis consolidated the patterns identified from the cluster analysis. The results from this study provide insight in making disease control and mitigation strategies.

## 1. Introduction

The novel coronavirus disease (COVID-19), first reported in Wuhan, China, presents a severe threat to global health [1,2]. The disease caused by the novel severe acute respiratory syndrome coronavirus 2 (SARS-CoV-2) is highly contagious and spreads rapidly [3]. The most common transmission pathway is through face-to-face exposure of expelled droplets during coughing, sneezing or talking [4]. Fever, cough and tiredness are common signs and symptoms of the infection [4]. The symptoms of patients with the disease can be mild or severe, even deadly [4]. The World Health Organization (WHO) declared the disease a global pandemic on 11 March 2020, due to its infectivity and severity. By 12 December 2020, the disease had infected more than 72 million people and led to over 1.6 million deaths worldwide.

Since the first case was reported in late January 2020, the United States (US) has been suffering the impact of COVID-19 severely [5]. Up to Mid-December 2020, the US reported more than 16 million cases, which is the highest in the world by country. The reported daily new cases vary greatly in space and time. Therefore, understanding the patterns of the disease occurrence is very important, because it helps to evaluate the effectiveness of mitigating strategies and policies in the past, and allocate new resources and implement updated control measures, as well as predict the future trend [6,7].

Recognizing a pattern is a process of discovering the regularities in data with statistical or machine learning algorithms and using these regularities to conduct further analysis such as clustering or classification [8]. Pattern recognition plays a vital role in exploring meaningful information and detecting complex relationships from a large data set, such as identifying risk factors and illustrating the trend of a disease [9,10]. Cluster analysis is one of the approaches to pattern recognition. It groups objects with similar attributes into the same cluster. Therefore, objects have high similarity within cluster while low similarity between clusters [11]. When applied to analyzing disease data, cluster analysis provides initial sights in clues to disease etiology and factors associated with diseases [12], such as geolocation, climate, social influence, and political preference. Identifying geospatial patterns of COVID-19 is also essential for disease mitigation because it helps to illustrate the extent and impact of the pandemic, develop public health policies and aid decision making and community action [13,14,15].

The objective of this study is to identify the patterns of the COVID-19 cases in the US. Specifically, we attempt to answer the following questions: What is the spatial pattern of COVID-19 cases? What is the trend of COVID-19 cases? What is the similarity of disease curves among these states? We applied geospatial analysis, time series analysis and K-means clustering analysis to identify these patterns.

## 2. Materials and Methods

### 2.1. Data Collection and Processing

We obtained the COVID-19 case data of the US from Johns Hopkins University [16], which are publicly assessable from the GitHub website [17]. These data are daily-county level data that started from 22 January 2020 to the present and are updated daily. Since the original dataset reports the number of cumulative cases each day, we calculated the number of new cases each day by subtracting the previous day’s case number from the current day’s case number.

Considering there were only very few cases before March 2020, we removed the data before 1 March 2020. Therefore, the data in our analysis start on 1 March and end on 12 December 2020. Spatially, we included the data from 50 states and the District of Columbia (DC).

We further generated two datasets from the cleaned dataset for different analyses. For spatial pattern analysis, we aggregated the data by the date to obtain the total number of cases in each county on the latest day of the study period. For K-means clustering and time-series analysis, we aggregated the data by state to obtain the daily new case data during the study period in each state.

### 2.2. Spatial Pattern Analysis

**Figure 1:**
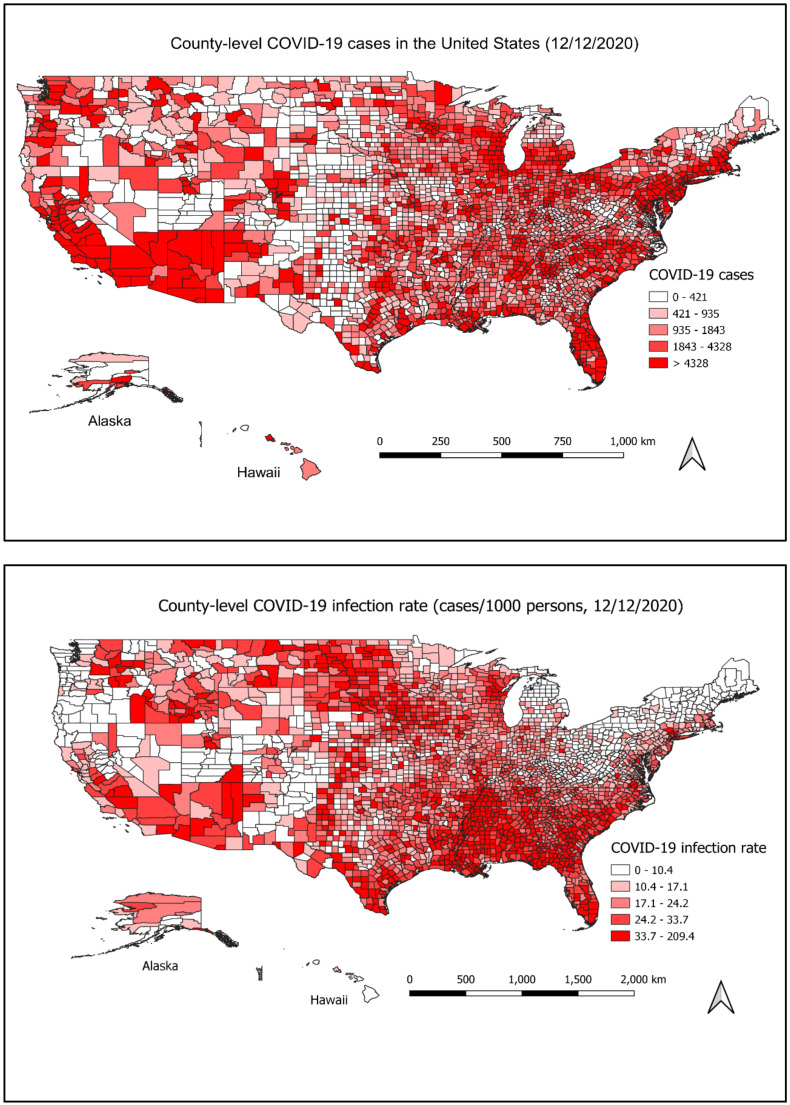
The county-level of COVID-19 cases and rates in the U.S on 12 December 2020. The map on the top shows the number of COVID-19 cases on 12 December 2020, in each county. The map on the bottom shows the infection rate of COVID-19 on 12 December 2020 in each county. The number of cases and the infection rate were divided into five categories and illustrated by red color from light (low number) to deep (high number), respectively.

To demonstrate the spatial distribution of COVID-19 cases, we mapped the total number of cases (up to 12 December) in each county with a geographic information system (GIS). We also calculated the rate of the disease using the total number of cases in each county divided by the population in that county. The county boundary GIS layer in 2018 was obtained from the US Census Bureau [18]. The disease data and the boundary layer were merged using the Federal Information Processing Standards (FIPS) county code (Figure 1).

We analyzed the spatial pattern of cases and rates of the disease using Global Moran’s I, which is an index to measure spatial autocorrelation based on the location and the target feature simultaneously [19,20]. Based on the index value, the pattern is determined as clustered, dispersed or random. We used The Spatial Autocorrelation (Global Moran’s I) tool in ArcGIS 10.1 (ESRI, Redland, CA, USA) to calculate the Moran’s I Index value. A z-score and *p*-value were also calculated to evaluate the significance of that Index.

### 2.3. Temporal Trend Analysis

To achieve a trend of daily cases in the US, we decomposed each time series data into three components: trend component, seasonal component and residual (random noise) [21]. The seasonal component is a pattern that repeats within a fixed period of time. We set the seasonality as seven days, considering that there is a weekly variation in daily new cases possible due to testing and reporting fluctuations [22]. We chose the additive model for the seasonal-trend decomposition.

Using the same method, we obtained the trend of COVID-19 cases for each state. Then, we plotted the trends of all states in descending order based on the total case number of each state on 12 December (see Table S1 in the Supplementary Materials). For better visualization, we put five states in a panel to plot their trends and modified the case number by the base-10 logarithmic transformation.

### 2.4. K-Means Clustering and Principal Component Analysis

We applied K-means clustering to explore the pattern of disease curves in different states. K-means clustering is a simple and common unsupervised machine learning algorithm for exploratory data analysis to get an intuition about data structure [8]. It divides these states into K subgroups (clusters). The states with a similar disease curve will be grouped into the same cluster. The K-means algorithm works as below: (1) Specify the number of clusters (K); (2) initialize K centroids and calculate the distance between each centroid and each data point; (3) the data points that have the shortest distance to a centroid are grouped in the same cluster; (4) calculate the new centroid based the data points in a cluster; (5) repeat the process from (2) to (4) until the centroids are stable [23]. To find an appropriate K value, we plot the total within-cluster sum of squares (WSS), namely, the total intra-cluster variation, against different K values and heuristically choose the number of clusters so that adding another cluster does not minimize the WSS [24].

**Figure 2:**
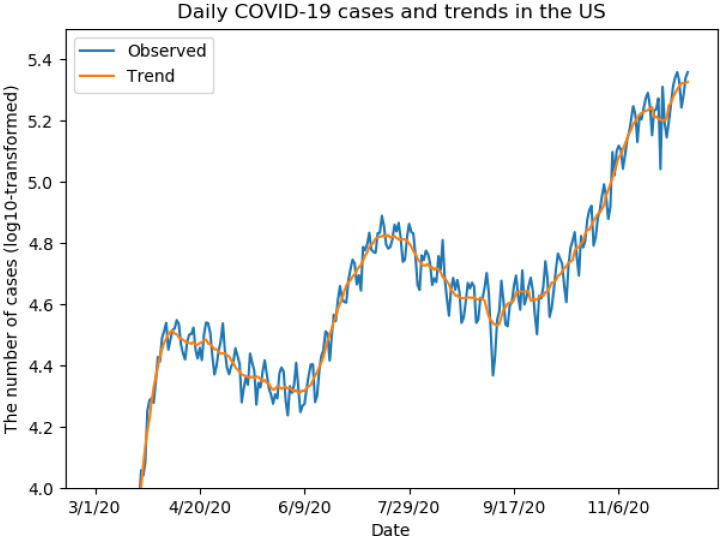
The trend of COVID-19 cases in the US during 1 March—12 December.

First, we conducted K-means clustering analysis for the whole period, then conducted the analysis for each of three phases, namely the early phase (1 March–31 May), the middle phase (1 June–30 September) and the late phase (1 October–12 December). Based on the trend analysis of COVID-19 daily cases in the US, we observed three main peaks in the whole study period (Figure 2). Therefore, we arbitrarily divided the whole period into three phases roughly. From the WSS plots, we selected the K value as three for the whole period analysis, and selected the K value as three, two, and four for each individual phase, respectively. We used k-means++ algorithm to initialize the centroids of clusters [25].

To illustrate these clusters and the similarity of the disease curve of each state, we further conducted a principal component analysis (PCA) on the same dataset. PCA is a common algorithm for dimensionality reduction. It projects each data point into a few principal components, which are computed by eigendecomposition of the covariance matrix of a dataset. The majority of variance of the data is preserved by the first few principal components [8,26].

By PCA, we converted high dimensional data (the daily new case data during the study period) to two-dimensional data (the first two principal components), which accounted for 87% of the total variance. Then we plotted the first two principal components use a 2-D scatterplot to identify the connection of these states in terms of daily case curves. Both K-means clustering and PCA were carried out using the “scikit learn” package from Python 3.7 [27].

## 3. Results

### 3.1. Spatial Distribution of COVID-19 Cases

The spatial distribution of the COVID case and rate in each county is shown in Figure 1. Up to 12 December, Los Angeles (California), Miami-Dade (Florida), Cook (Illinois), Maricopa (Arizona) and Harris (Texas) are the top five counties with the most total cases, of which the total number of cases is above 200,000. Trousdale (Tennessee), Lincoln (Arkansas), Chattahoochee (Georgia), Lafayette (Florida) and Lake (Tennessee) are the top five counties with the highest rate, of which the rates are above 100 cases/1000 persons.

Spatial autocorrelation analysis indicates that the number of cases was clustered at the county level. The result showed the global Moran’s I is 0.0264, z-score is 22.7343, and *p*-value < 0.0001. The county-level COVID-19 rates were also spatially clustered (Global Moran’s I = 0.2288, z-score = 185.4885, *p*-value < 0.0001).

### 3.2. Temporal Trend of COVID-19 Cases

As shown in Figure 2, the case number in the US increased rapidly in mid-March and reached the first peak in early April. After that, the number of cases decreased gradually until early June, when the number increased again. The second peak appeared in late July, and the case number went down gradually until mid-September. After that, the number uprose dramatically, particularly in November and December.

**Figure 3:**
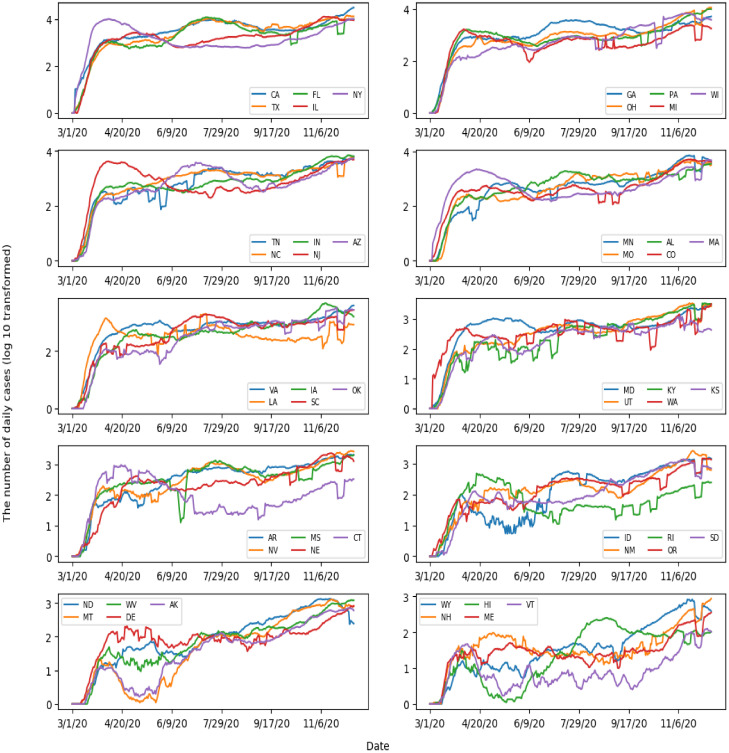
Trends of Covid-19 cases in 50 states during 1 March–12 December. The name and abbreviation of each state is listed as below: California (CA), Florida (FL), New York (NY), Texas (TX), Illinois (IL), Georgia (GA), Pennsylvania (PA), Wisconsin (WI), Ohio (OH), Michigan (MI), Tennessee (TN), Indiana (IN), Arizona (AZ), North Carolina (NC), New Jersey (NJ), Minnesota (MN), Alabama (AL), Massachusetts (MA), Missouri (MO), Colorado (CO), Virginia (VA), Iowa (IA), Oklahoma (OK), Louisiana (LA), South Carolina (SC), Maryland (MD), Kentucky(KY), Kansas (KS), Utah (UT), Washington (WA), Arkansas (AR), Mississippi (MS), Connecticut (CT), Nevada (NV), Nebraska (NE), Idaho (ID), Rhode Island (RI), South Dakota (SD), New Mexica (NM), Oregon (OR), North Dakota (ND), West Virginia (WV), Alaska (AK), Montana (MT), Delaware (DE), Wyoming (WY), Hawaii (HI), Vermont (VT), New Hampshire (NH), and Maine (ME).

The trends of daily cases in 50 states are illustrated in Figure 3 and Figure S1 in the Supplementary Materials. In the early phase (1 March–31 May), the disease curves in New York, New Jersey, and Massachusetts stood out, of which the case numbers were higher than other states. The disease curves of Texas and Florida were very similar. Before June, the number of new cases was very low. However, the case number increased quickly and formed a large peak in mid-July and gradually decreased in August and September. The curve of California was similar to that of Florida and Texas in the early and the middle phases (1 March–30 September). However, the number of cases increased dramatically after October. Interestingly, the temporal trends for Alaska, Montana and Hawaii are quite different from other states, where they had a dramatic decrease in case numbers in the summer. The curves in Connecticut and Rhode Island are also worth attention. In both states, the main peak appeared in the early phase, but the numbers of cases kept lower in the middle and late phases.

### 3.3. K-Means Clustering and PCA

**Figure 4:**
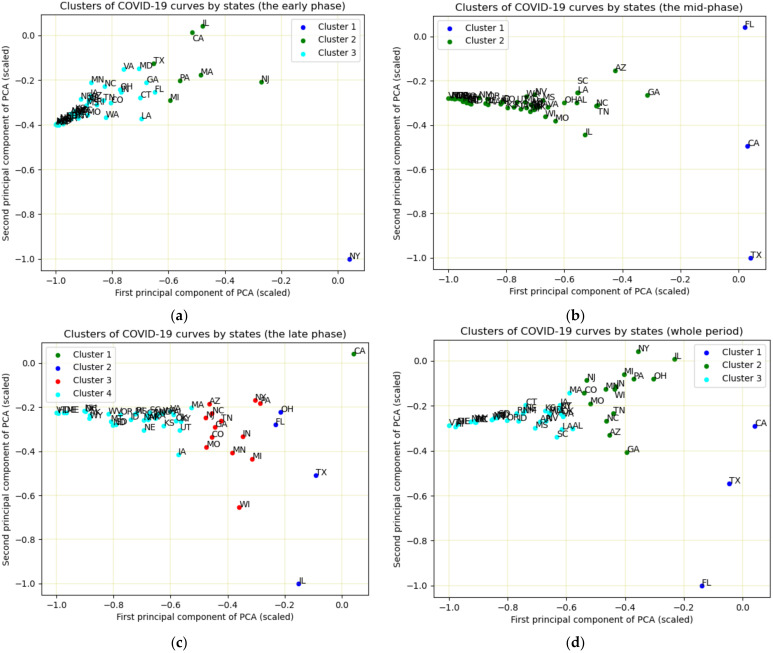
K-means clustering and PCA of COVID-19 curves in each state in the early phase (**a**), the middle phase (**b**), the late phase (**c**) and the whole period (**d**).

The results of K-means clustering and PCA were displayed in Figure 4 and Table 1. In the early phase (1 March–31 May), three clusters were obtained. New York had a unique pattern of disease curve and was assigned in one cluster alone (Figure 4a). California, Illinois, Massachusetts, Michigan, New Jersey, Pennsylvania and Texas were assigned into the second cluster. The remaining states were assigned into one cluster. The PCA scatter plot (Figure 4a) showed that New York, located at the right-bottom, is far away from other states. The nearest state to New York in the scatter plot was New Jersey, which had a similar disease curve as New York. In the second cluster, California and Illinois were very close, but far away from other states. The states in the three clusters were concentrated in the left-center.

Two clusters were observed in the middle phase (1 June–30 September). California, Texas and Florida were assigned in the same cluster, which had a pattern much different from the remaining states. In the PCA scatter plot (Figure 4b), Florida, California and Texas were close to the right boundary, while Florida was located at the top right, California and Texas were located at the bottom right. The remaining states were assigned to one cluster and located on the left-center.

In the late phase (1 October–12 December), California had a unique pattern of the disease curve and was assigned a cluster alone (Figure 4c). Texas, Illinois, Ohio and Florida were assigned in the second cluster. New York, Pennsylvania, Arizona, New Jersey, North Carolina, Tennessee, Georgia, Colorado, Missouri, Indiana, Minnesota, Michigan and Wisconsin were assigned in the third cluster, and the remaining states were assigned into the fourth cluster.

In the whole period, three clusters were observed. California, Texas and Florida had similar patterns and were assigned in the same cluster. Arizona, Colorado, Georgia, Illinois, Indiana, Michigan, Minnesota, Missouri, New Jersey, New York, North Carolina, Pennsylvania, Tennessee and Wisconsin were assigned in the second cluster, and the remaining states were classified into the same cluster. In the PCA scatter plot (Figure 4d), Florida was located at the bottom left, which was far away from the other states. California and Texas were located at the left-center.

## 4. Discussion

In this study, we explored the patterns of COVID-19 cases in the US from 1 March to 12 December 2020. First, we illustrated how the number of COVID-19 cases varied in space and time. Then we examined the similarity of the disease curves from different states. To our knowledge, this is the first study to investigate the patterns of the COVID-19 pandemic in the US using K-means clustering and PCA.

The GIS mapping shows COVID-19 cases at the county level were spatially clustered, which is consistent with the findings from other studies [13,28]. The map shows the higher rates and higher number of cases in urban areas, which might be because that the population in the metropolitan area is much more condensed than that of rural areas, and many high-risk populations such as minorities, and older adults live in metropolitan or urban areas [29]. The clustering suggests that it might make sense for some states to take a county-by-county approach for re-opening rather than a one-size fit all policy to curb the fast spread of the disease in areas with a high population density. However, it should be cautious in adopting the county-by-county policy as the situation can change. For example, California was adopting a county-by-county policy in COVID-19 mitigation. However, in November, the disease was spreading fast in every corner, including both high-density and less-density counties [30].

Trend analysis shows that the pandemic in the US is deteriorating, and the number of daily cases is still very high. Though there are a lot of variations in the number of daily cases, we observed three main peaks. In the early phase, New York, New Jersey, Massachusetts and Washington were severely affected by the virus. In the middle phase, Florida, Arizona, Texas and California reported a large number of cases, which were the major contributors to the second peak of the disease curve in the US. In the late phase, California becomes the most affected state by the pandemic, and many states reported the rapid increase in new cases, such as Ohio, Pennsylvania, Michigan and Illinois. Only a few states showed that the number of daily cases was lower in the middle phase and late phase than in the early phase, such as Connecticut, which might suggest that the pandemic is well handled in these states.

Using K-means and PCA, we not only revealed which states were clustered together in terms of the disease curve but also illustrated the degree of similarity of the disease curve in each state. In the scatter plot of the first two principal components, each data point represents a state. The close data points indicate that these states have similar disease curves. There are several reasons to explain the similarity of the disease curves. First, the states are geographically connected, and people may frequently commute between states, for example, North Carolina and Tennessee. Second, the states implement similar disease control measures. For example, Both Texas and Florida were early in reopening the state’s economy. Third, people may have similar awareness about COVID-19 and take active self-protection measures (e.g., mask-wearing) [31].

The pattern of the disease curve might be potentially related to the mitigation policy in its state, such as New York, Texas, Florida and California. The disease curve in New York has a unique pattern in the early phase (1 March–30 May). The number of cases in New York started to jump in March and peaked in April and then dropped with a flat curve since June. During that period, New York City (NYC), the area with a dense population, was an epicenter of COVID-19 [32]. To respond to the health crisis, New York state issued a stay-home-order on non-essential business closures, large gathering banns in March, and then take a phased approach for reopening [33]. In the mid-June, most indoor activities such as in-door dining for restaurants and bars were still prohibited [34]. These policies might result in the flat curve of the disease in the middle phase.

The patterns of the disease curve in California, Texas and Florida were very similar in the middle phase. The cases increased slowly before June and peaked in July, then decreased in August and September. The peak time was about two or three weeks after Florida and Texas entered a phase to reopen their business, respectively. Different from New York, during this period, indoor activities for the three states were permitted. For example, bars and restaurants were allowed to open at 50% capacity in Florida. When the relaxation criteria for counties for reopening were announced on May 18, California also allowed indoor activities. For example, houses of worship could open to 100 people, or at 25% capacity [35]. These factors might contribute to the high number of COVID-19 cases during that period. Texas and Florida paused reopening and reversed the course of restriction at the end of June [36], and the California governor had to order rollback of restrictions effective on July 13 [35]. After the rollback, we observed the decrease of case numbers for the three states.

Comparing New York with these three states, we found that New York was at a slow pace in regard to reopening and the rules were stricter for high-risk businesses such as restaurants and bars. In addition, both Florida and Texas ordered schools to open for fall, schools in California were open on a district-by-district basis [37]. However, New York did not order for schools to re-open. The consistency between the results of the pattern analysis and state reopening policy suggested mitigation strategies greatly influenced the spread of COVID-19 in different states.

We observed a dramatic decrease in the number of COVID-19 cases in Alaska, Montana and Hawaii in the summer. This might have resulted from mitigation policies implemented in these states. For example, in Alaska, the Municipality of Anchorage (Alaska’s largest city) issued emergency orders (EOs) requiring facial mask covering in most public locations. After these EOs took effect, self-reported mask-wearing increased and the number of cases dropped, which suggested that these mitigation policies were effective in slowing down the transmission of the disease [38].

The cases in most states were rising in the late phase (1 October–12 December). California had a unique pattern in this period, which demonstrated a surge without a sign of decreasing. The surge of COVID-19 not only affects major urban areas, such as Los Angeles, but also the far northern rural areas. A large proportion of those cases were people who were gathering indoors [31]. In contrast, Texas, Illinois and Ohio showed increasing cases at the beginning of November, a peak around the Thanksgiving holiday, and then the cases decreased after Thanksgiving. New York, Pennsylvania, Arizona, New Jersey, North Carolina, Tennessee, Georgia, Colorado, Missouri, Indiana, Minnesota, Michigan and Wisconsin were characterized by a stair-shaped rise in cases from the end of October and the beginning of November. The unanimous increasing cases in this period might be due to multiple reasons. First, most respiratory diseases and viruses spread faster in cool and cold weather. Second, many events happened in November, such as the presidential election and the Thanksgiving holiday. It is likely that many people gathered together regardless of the state warnings. Third, COVID-19 caution fatigue can be another reason for the dramatic increase [39]. After more than half a year, people may become less motivated in taking personal safety precautions.

Not all states are facing the surge of the COVID-19 cases in the late phase, such as Connecticut. The curve of Connecticut had been stable until October. Upon examining its mitigating policy, we found that Connecticut was slower in reopening compared to many of the other states, and the bars kept closed. In addition, the governor issued strict facial covering rules as early as 17 April [40,41] These policies might play a positive role in mitigating the disease.

The findings from this study have a few public health implications. First, we analyzed the spatial pattern of COVID-19 and showed areas with high rates of the disease, which can help public health professionals to target specific hotspot areas and implement intervention strategies effectively. Second, we illustrated the trend of the disease for each state, which can help to evaluate the effectiveness of the mitigation measures and understand the spread of the disease in the following days. Finally, we analyzed the pattern of the disease curve using cluster analysis. Identifying the common or discriminative factors among the clusters is important for making effective mitigation policies or evaluating the effectiveness of mitigation policies. For example, California, Texas and Florida were grouped in the same cluster in the middle phase. In these states, indoor activities were allowed, the social distance was difficult to keep given their large population.

This study has a few limitations. First, we only obtained COVID-19 data at the county level. Therefore, we cannot explore clusters at smaller spatial units. Second, K-means clustering has its own weakness. For example, the linear separability of data and the selection of K value are likely to influence the results of clustering. Finally, we did not investigate risk factors or covariates for COVID-19. Therefore, it is difficult to accurately interpret the patterns observed. Further studies to explore factors associated with the pattern of the diseases are needed.

## 5. Conclusions

The pattern of disease curves among 50 states varied in different time periods. In the early phase (1 March–31 May), three clusters were observed, and New York had a unique pattern of the disease curve. Two clusters were observed in the middle phase (1 June–30 September), of which California, Texas and Florida were assigned in the same cluster. In the late phase (1 October–12 December), four clusters were observed, and California had a unique pattern of the disease curve. The trends of disease curves from seasonal-trend composition consolidated the results obtained from the cluster analysis. The information is useful for understanding the spread of the disease and make appropriate mitigation strategies.

## Figures and Tables

**Table 1:** The results of K-means cluster analysis in different time periods.

Time Period	Clusters	States
The early phase (1 March–31 May)	1	NY
	2	CA, IL, MA, MI, NJ, PA, TX
	3	The remaining states
The middle phase (1 June–30 September)	1	CA, FL, TX
	2	The remaining states
The late phase (October–12 December)	1	CA
	2	FL, IL, OH, TX
	3	AZ, CO, GA, IN, MI, MO, MN, NC, NJ, NY, PA, TN, WI
	4	The remaining states
The whole period (1 March–12 December)	1	CA, FL, TX
	2	AZ, CO, GA, IL, IN, MI, MN, MO, NC, NJ, NY, OH, TN, WI
	3	The remaining states

## Data Availability

The data used in the study is public accessible from the website: https://github.com/CSSEGISandData/COVID-19.

## Supplementary Materials

The following are available online at https://www.mdpi.com/1660-4601/18/5/2493/s1, Table S1: The number of COVID-19 case in each state of the US by 12 December 2020, Figure S1: Trends of Covid-19 cases in 50 states during 1 March–12 December 2020 using untransformed data.

## Code Availability Statement

The code for analyzing the pattern of COVID-19 is available from the website https://github.com/science21/Pattern-recognition-of-COVID-19-Pandemic.
