# Supplementary material for: Pattern Recognition of the COVID-19 Pandemic in the United States: Implications for Disease Mitigation
Source: Int J Environ Res Public Health. 2021 Mar 3;18(5):2493. doi: 10.3390/ijerph18052493 (PMC7967616; doi:10.3390/ijerph18052493)
Supplement: Supplementary file 1 [file ijerph-18-02493-s001.pdf]

Table S1. The number of COVID-19 case in each state of the US by December 12, 2020.

State	Abbreviation	Cases
California	CA	1550914
Texas	TX	1414437
Florida	FL	1120014
Illinois	IL	851796
New York	NY	766441
Georgia	GA	566401
Ohio	OH	555172
Pennsylvania	PA	486284
Michigan	MI	470148
Wisconsin	WI	468469
Tennessee	TN	454571
North Carolina	NC	432243
Indiana	IN	420586
New Jersey	NJ	413543
Arizona	AZ	402919
Minnesota	MN	378481
Missouri	MO	352524
Alabama	AL	292848
Colorado	CO	286830
Massachusetts	MA	286385
Virginia	VA	279917
Louisiana	LA	275614
Iowa	IA	256966
South Carolina	SC	249399
Oklahoma	OK	234795
Maryland	MD	232644
Utah	UT	232091
Kentucky	KY	222437
Washington	WA	203604
Kansas	KS	189809
Arkansas	AR	189218
Nevada	NV	185193
Mississippi	MS	178995
Nebraska	NE	149133
Connecticut	CT	148785
Idaho	ID	121850
New Mexica	NM	120528
Rhode Island	RI	113234
Oregon	OR	93006
South Dakota	SD	90615
North Dakota	ND	87836
Montana	MT	73165
West Virginia	WV	62553
Delaware	DE	45633
Alaska	AK	40522
Wyoming	WY	39220
New Hampshire	NH	31167
Hawaii	HI	19602
Maine	ME	15943
Vermont	VT	5891

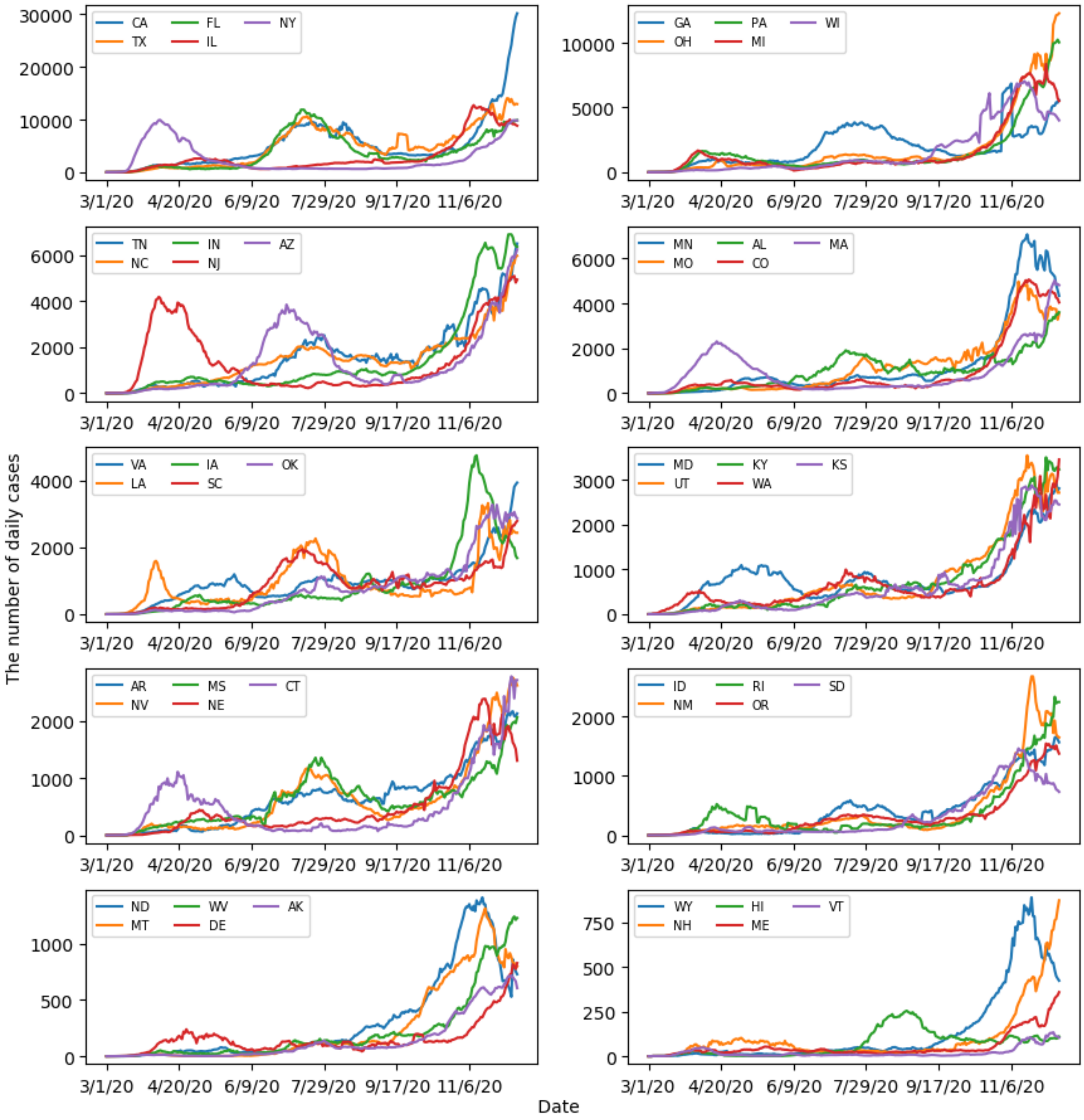

Figure S1. Trends of Covid-19 cases in 50 states during March 1-December 12, 2020 using untransformed data.
